# Hepatoprotective effect of juglone on dimethylnitrosamine-induced liver fibrosis and its effect on hepatic antioxidant defence and the expression levels of α-SMA and collagen III

**DOI:** 10.3892/mmr.2015.3992

**Published:** 2015-06-24

**Authors:** DE-JIANG ZHOU, DONG MU, MING-DE JIANG, SHU-MEI ZHENG, YONG ZHANG, SHENG HE, MIN WENG, WEI-ZHENG ZENG

**Affiliations:** Internal Medicine Department of Digestion, General Hospital of Chengdu Military Region, Chengdu, Sichuan 610083, P.R. China

**Keywords:** liver fibrosis, juglone, immunohistochemical staining, oxidative stress, α-smooth muscle actin

## Abstract

The present study aimed to investigate the antifibrotic effects of juglone on dimethylnitrosamine (DMN)-induced fibrosis in rats. Juglone, which is a quinone, significantly decreased DMN-induced rat hepatic fibrosis, which was associated with increased superoxide dismutase (SOD) activity, decreased oxidative stress and reduced levels of α-smooth muscle actin (α-SMA) and collagen (Col) III in the liver. Serum levels of alanine aminotransferase, aspartate aminotransferase, hyaluronic acid, laminin, type III precollagen and type IV collagen were significantly reduced by treatment with juglone. Liver fibrosis was induced in male Sprague-Dawley rats by subcutaneous injections of DMN solution and hepatic fibrosis was assessed using Massonstrichome staining. The expression levels of α-SMA and Col III were determined using immunohistochemical techniques. The activities of SOD and malondialdehyde in liver homogenates were also determined. The results suggested that juglone augmented the antioxidative capability of the liver, possibly by stimulating the activity of SOD, which promoted the inactivation of hepatic stellate cells (HSCs) and decreased the accumulation of extracellular matrix collagen in the liver, thereby alleviating hepatic fibrosis. Silymarin was used as a positive control for liver fibrosis protection. It was hypothesized that juglone alleviates or mitigatesoxidative stress-mediated hepatic fibrosis by upregulating the expression of peroxisome proliferator-activated receptor γ and inhibiting the activation of HSC.

## Introduction

Liver fibrosis results from chronic damage to the liver in conjunction with the accumulation of extracellular matrix (ECM) proteins, including collagen, which also occurs in the majority of types of chronic liver disease (1). Advanced liver fibrosis results in cirrhosis, liver failure and portal hypertension, and often requires liver replacement. The build upof ECM proteins distorts hepatic morphology by developing a fibrous scar, and the consequent development of nodules of regenerating hepatocytes describes cirrhosis (2). Cirrhosis produces hepatocellular dysfunction and amplified intrahepatic opposition to blood flow, which results in hepatic inadequacy and portal hypertension, respectively (3). Hepatic fibrosis occurs as the result of the continued wound healing response of the liver to toxic, infectious or metabolic agents. Chronic hepatitis C infection, alcohol abuse and non-alcoholic steatohepatitis are also major causes of hepatic fibrosis (4–5). Early clinical reports in the 1970s suggested that advanced liver fibrosis is reversible (6), however, in the 1980s, hepatic stellate cells (HSCs), formerly known as lipocytes or peri sinusoidal cells, were recognized as the predominant collagen-producing cells in the liver (7). This cell type undergoes marked phenotypic activation in chronic liver disease, with the acquisition of fibrogenic properties (8). Activation of HSCs leads to retinoid storage, remodeling of the ECM and the production of growth factors and cytokines (9,10). Suppression of HSC activation has been suggested as a therapeutic target against hepatic fibrosis (11–13).

Oxidative stress is a key pathogenic factor in several types of liver disease, which can cause hepatocyte destruction through lipid peroxidation and protein alkylation. Superoxide dismutase (SOD) and catalase are central antioxidant enzymes, which function as endogenous free radical scavengers (14). However, previous studies have identified metallothionein(MT) as a more competent scavenger for reactive oxygen species (ROS) (15,16).

Quinones in the plant kingdom represent a broad category of widely distributed quinoid compounds in nature. Several quinones have been associated with a wide range of bioactivities. At present, a number of clinically important pharmaceutical agents containing a quinone nucleus with significant anticancer activity have been confirmed, including anthracycline, mitoxantrones and saintopin. These quinoid compounds predominantly target DNA, however, the exact contribution of quinone moiety to the anticancer effect remains to be fully elucidated. In general, quinone toxicity is attributed to the ability to undergo reversible oxidation-reduction reactions and to its electrophilic nature, leading to the formation of free radicals (17). Juglone, also termed 5-hydroxy-l,4-naphthalenedione, belongs to the quinoid family of compounds and occurs naturally in the leaves, roots, husks, fruit and bark of plants in the Juglandaceae family, in particular, the black walnut (*Juglansnigra*) (18). Juglone is an example of an allelopathic compound, which is a substance that is synthesized by one type of plant and affects the growth of another (19–22).

At present, there are no effective drugs to avert or treat liver fibrosis, and certain natural products with antioxidant potential are being investigated for developing novel therapeutic reagents. In view of the lack of effective remedial measures for hepatic fibrosis, the present study aimed to investigate the antifibrotic effects of jugalone, and established that treatment of rats with juglone reduced DMN-induced hepatic fibrosis. The present study also demonstrated that jugalone increased the activity of SOD and decreased oxidative stress in the liver, suggesting that liver fibrosis protection by juglone may occur by increasing the antioxidative capability of the liver. It was also observed that serum levels of alanine aminotransferase (ALT), aspartate amino-transferase (AST), hyaluronic acid (HA), laminin (LN), type III procollagen (PCIII) and type IV collagen (CIV) were significantly reduced by treatment with juglone. The effect of juglone on the expression levels of α-smooth muscle actin (α-SMA) and collagen (Col) III in the liver were also examined, which revealed that juglone significantly reduced the expression levels of α-SMA and Col III in the liver.

## Materials and methods

### Chemicals and animal treatments

Juglone was purchased from the Shandong Engineering Research Center for Natural Drugs (Yantai, China), the purity of which was 99.5%, according to high-performance liquid chromatography (acetonitrile: water, 90:10; C_18_ column). Analytical grade dimethylnitrosamine (DMN) was purchased from Chengdu Kelong Chemical Reagent Factory (Chengdu, China) and silymarin was purchased from Tianshili Pharmaceutical Company (Tianjing, China). A total of 30 male Sprague-Dawley rats, weighing 150±25 g, were obtained from the Experimental Animal Center of Guiyang Medical College (Guiyang, China; Approval no. SCXK 2001–0022). The present study was performed according to instructions approved by the Institutional Ethical Committee of the General Hospital of Chengdu Military Region (Chengdu, China). The rats were randomly divided into the following five groups, each containing six rats: Controlgroup, DMN-induced hepatic fibrosis (model) group, juglone prevention (JP) group, silymarin prevention (SP) group and juglone + silymarin prevention (JP+SP) group. The rats in the model, JP, SP and JP+SP groups received subcutaneous injections of DMN solution at a dose of 0.5 ml/kg twice weekly for 4 weeks, with an initial dose of 0.2 ml/kg. These rats were fed a high-lipid/low-protein diet. The rats in the control group were fed a normal diet. Juglone (200 mg/kg), silymarin (1.0 g/kg) and juglone (200 mg/kg) + silymarin (1.0 g/kg), were administered once a day by gastric gavage to the rats in the In the JP, SP and JP+SP groups, respectively. After 4 weeks, the rats were sacrificed using anesthesia (anaestheticpropofol, 0.1 ml/100 mg) and a midline incision was made to remove the liver; ~2 ml blood was collected from each rat, as well as 0.5–1 mm liver lobe sections. The same section of each liver was removed and fixed in 10% neutral formalin (Sigma-Aldrich, St. Louis, MA, USA). The residual portion of liver was stored at −50°C. Rat serum was prepared by centrifugation at 224 x g for 15 min at room temperature and stored at −50°C.

### Biochemical parameters for the assessment of liver function

The serum levels of ALT, AST, HA, LN, PCIII and CIV were measured using commercially available kits as follows: Alanine Aminotransferase BioAssay™ ELISA kit, Aspartate Aminotransferase BioAssay™ ELISA kit, Hyaluronic Acid BioAssay™ ELISA kit, LamininBioAssay™ ELISA kit, Procollagen III BioAssay™ ELISA kit and Collagen Type IV (CIV) BioAssay™ ELISA Kit (Wuhan Boster Biological Engineering Co., Ltd., Wuhan, China) and were performed, according to the manufacturer's instructions.

### SOD and malondialdehyde (MDA) in liver tissue

The tissue samples were homogenized in cold 20 mM HEPES buffer, containing 1 mM EGTA, 150 mMmannitol and 30 mM sucrose (pH 7.1; all Sigma-Aldrich), using a tephlon homogenizer (Polytron RZR 1; Heidolph, Schwabach, Germany). The cell debris was detached by centrifugation at 2,000 × g for 5 min at 4°C. The supernatants were used for the assessment of SOD activity, with assays conducted according to the manufacturer's instructions (Cayman Chemical Company, Ann Arbor, MI, USA). The levels of MDA were also estimated using a thiobarbituric acid method, according to the manufacturer's instructions (Cayman Chemical Company).

### Histopathology of liver tissues

Following fixation in 10% formalin for 24 h, the liver samples were embedded in paraffin (Sigma-Aldrich). The samples were then cut into 5-mm tissue sections and mounted onto slides (Sigma-Aldrich). The sections were processed for hematoxylin and eosin (H&E) and Masson's trichrome (both Sigma-Aldrich) staining for assessment of the degree of liver fibrosis. A second set of tissue sections (size, 0.5×0.5 cm) were prepared for immunohistochemical assessment of the tissues and mitotic indexing (MI). The liver tissues were further assessed for histopathological examination by a single observer in a blinded-manner.

### Immunohistochemical analysis and MI

The liver sections were incubated in 2% H_2_O_2_ (Qingdao HiseaChem Co., Ltd., Qingdao, China) for 15 min following deparaffinization in xylene, rehydration in graded ethanol and antigenunmasking by heat treatment using citrate buffer. The sections were subsequently incubated with mouse anti-α-SMA (cat. no. SC-32251; Santa Cruz Biotechnology, Inc., Santa Cruz, CA, USA), mouse anti-Col III (cat. no.SC-80564; Santa Cruz Biotechnology) and rabbit anti-MT (cat. no.SC-11377; Santa Cruz Biotechnology, Inc.)antibodies (all at 1:500 dilution) overnight at 4°C Anti-mouse IgG in rabbit (cat no. M7023; 1:500; Sigma-Aldrich) was used as the secondary antibody. The samples were subsequently processed using an EnVision kit (Dako Denmark A/S, Glostrup, Denmark), according to the manufacturer's instructions. Phosphate-buffered saline (Sigma-Aldrich) served as a negative control. The cells with brown staining in the cytoplasm/nucleus were considered to be positive. A total of five high power microscopic fields (magnification, x500) were randomly selected in each slide and the numbers of positive cells in each field were counted under a microscope (Eclipse E600; Nikon Corporation, Tokyo, Japan (α-SMA and Masson's trichromeimmunopositivity). For the Col III staining, five high power fields were randomly selected in each tissue section and images were captured using a BioMias 2000 image analysis instrument (Image and Figure Research Institute, Sichuan University, China), to quantify the transparency of the immune-positive areas in the tissue section.

Cell proliferation was assessed by counting the number of mitotic cells per high-power field at a magnification of x200. For each slide, 10 randomly selected fields were counted. The MI was defined as the number of mitotic cells per 1,000 hepatocytes in paraffin-embedded liver samples stained with H&E.

### Western blot analysis

The liver tissues were treated with TRIzol protein extraction reagent (Invitrogen Life Technologies, Carlsbad, CA, USA), according to the manufacturer's instructions, and the protein concentrations were determined using Lowry's method (23). The protein samples were separated using 10% sodium dodecyl sulfate-polyacrylamide gel electrophoresis (Sigma-Aldrich) and electroblotted onto polyvinylidenedifluoride membranes (Millipore Corporation, Billerica, MA, USA). Following blocking with 1.5% bovine serum albumin (Sigma-Aldrich), the membranes were incubated overnight at 4°C with primary antibodies against peroxisome proliferator-activated receptor (PPAR)-γ (mouse anti-PPAR-γ; cat. no. SC-7273), α-SMA (mouse anti-α-SMA; cat. no.SC-32251), TGF-β1 (rabbit anti-TGF-β1; cat. no.SC-146), TIMP-1 (rabbit anti-TIMP-1; cat. no.SC-5538) and MMP-2 (rabbit anti-MMP-2; cat. no. SC-10736) obtained from Santa Cruz Biotechnology, Inc. The membranes were further incubated with secondary antibody for 1 h at room temperature. The protein bands were detected using an enhanced chemiluminescence detection system (Pierce Biotechnology, Inc., Rockford, IL, USA) and the protein expression levels were determined using Quantity One software version 4.5 (Bio-Rad Laboratories, Inc., Hercules, CA, USA).

### Statistical analysis

Statistical analyses were performed using one-way analysis of variance followed by Bonferroni's post-hoc test, using SPSS 20.0 software (SPSS, Inc., Chicago, IL USA). P<0.05 was considered to indicate a statistically significant difference.

## Results

### Juglone treatment decreases the activity of functional liver enzymes

**Figure 1:**
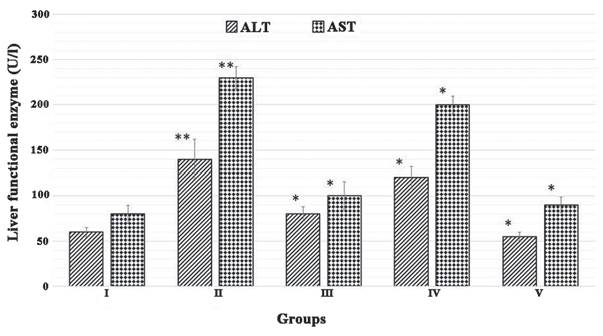
Juglone treatment decreases the activity of functional liver enzymes. Group I, normal control; Group II, model control + dimethylnitrosamine; Group III, 200 mg/kg silymarin treatment; Group IV, 200 mg/kg juglone treatment; Group V, 400 mg/kg juglone treatment. The results were analyzed using one-way analysis of variance, followed by Tukey's post-hoc test. The data are expressed as the mean ± standard deviation (n=10; **P<0.01, vs. normal control group; *P<0.01, vs. model control). ALT, alanine amino-transferase; AST, aspartate amino-transferase.

The effect of juglone treatment on functional liver enzymes is shown in Fig. 1. Biochemical analysis revealed that the DMN-treated rats exhibited a marked increase in the serum levels of ALT and AST, which were significantly higher, compared with those in the normal rats (P<0.01; n=10). However, the increased levels of these liver enzymes were efficiently reduced by treatments with silymarin and juglone (P<0.01; n=10). Juglone significantly restored the biochemical parameters to levels comparable with those following treatment with the silymarin, used as a standard drug in the present study.

### Juglone treatment inhibits DMN-induced collagen accumulation

**Figure 2:**
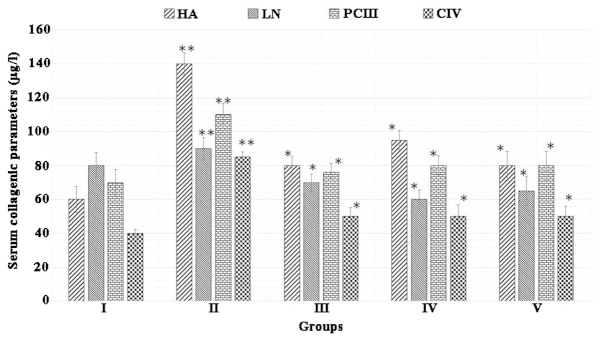
Juglone treatment reduces the accumulation of collagen. Group I, normal control; Group II, model control + dimethylnitrosamine; Group III. 200 mg/kg silymarin treatment; Group IV, 200 mg/kg juglone treatment; Group V, 400 mg/kg juglone treatment. The results were analyzed using one-way analysis of variance, followed by Tukey's post-hoc test. The data are expressed as the mean ± standard deviation (n=10; **P<0.01, vs. normal control, *P<0.01, vs. model control). HA, hyaluronic acid; LN, laminin; PCIII, type III procollagen; CIV, type IV collagen.

The results of the effect of juglone on collagenic accumulation in the livers of rats are shown in Fig. 2. The results demonstrated that, in rats treated with DMN (the liver fibrosis model) significantly increased serum levels of HA, LN, PCIII and CIV were observed, compared with the control (P<0.01; n=10). However, following treatment with silymarin and juglone, a gradual decrease in the activities of HA, LN, PCIII and CIV were observed in the fibrotic tissues (P<0.01, vs. model group; n=10). These results suggested that treatment with juglone inhibited DMN-induced collagenic changes in these animals. The effect of juglone effect was more marked, compared with that of silymarin, which was used as the standard.

### Effect of juglone on SOD and MDA contents of liver

**Figure 3:**
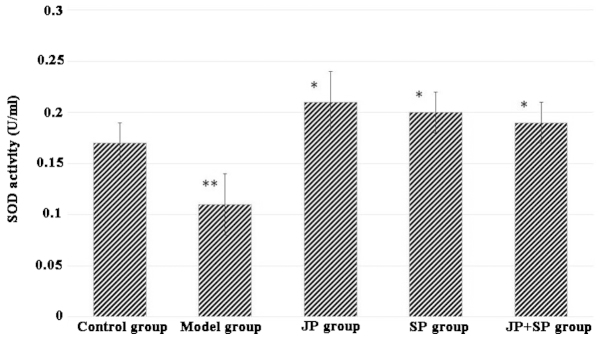
Effect of juglone on the activity of SOD in the hepatic tissues. The data are expressed as the mean ± standard error of the mean (n=6/group). Data revealed a significant difference between the JP, SP and JP+SP groups and the dimethylnitrosamine control group (*P<0.05) and between the model and normal control group (**P<0.05). SOD, superoxide dismutase; JP, juglone treatment; SP, silymarin treatment.

**Figure 4:**
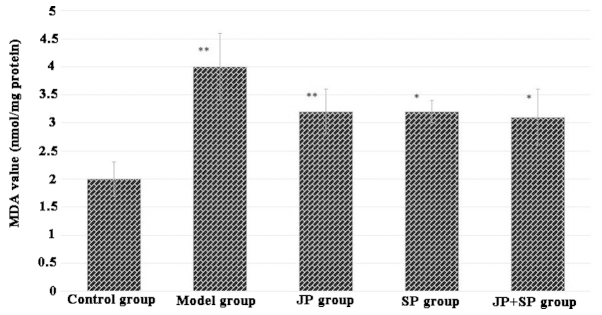
Effect of juglone on the level of MDA in the liver tissue. The data are expressed as the mean ± standard error of the mean. (n=6 /group) Significant differences were observed between the JP, SP and JP+SP groups and the dimethylnitrosamine control group (*P<0.05) and between the model group and the control group (**P<0.05). MDA, malondialdehyde; JP, juglone treatment; SP, silymarin treatment.

The antioxidant activity of the DMN group indicated that SOD was significantly reduced compared with the normal group. The JP group or SP group significantly restored the SOD level (Fig. 3). MDA level of liver homogenate was significantly high in the model group (DMN-treated group), however, administration of juglone or silymarin significantly reduced the level of MDA. The results of JP group were comparable with the SP group (Fig. 4). The combination of juglone and silymarin demonstrated a significant effect on the decrease of MDA in rat liver homogenate.

### Effects of juglone treatment on liver injury and morphology

**Figure 5:**
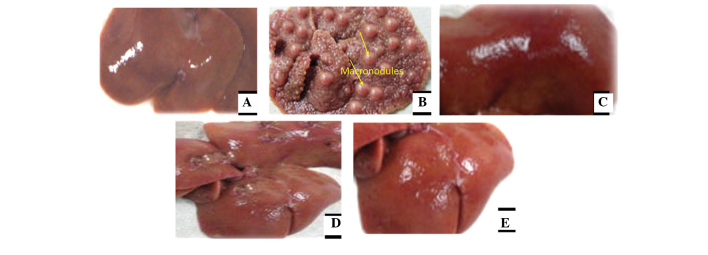
Morphological analysis of the effects of juglone on DMN-induced liver damage in rats. (A) Normal control group exhibited a normal and even surface. (B) DMN control group exhibited numerous macronodules in the liver. (C) Tissues from rats treated with DMN + silymarin exhibited a normal even surface. (D) Tissues from rats treated with DMN + 200 mg/kg juglone exhibited marginal damage. (E) Tissues from rats treated with DMN + 400 mg/kg juglone exhibited a smooth and even surface. Higher doses of juglone preserved the normal liver anatomical shape and appearance. DMN, dimethylnitrosamine.

The appearance of the liver tissues in each group are shown in Fig. 5. It was evident that, with the exception of the DMN-treated group, which exhibited considerable nodules and irregularities, the liver tissues in all the groups generally exhibited smooth and even surfaces with no signs of nodules. Furthermore, the normal group exhibited normal growth, whereas the rats in the DMN-treated model group exhibited lower body weights. The protective effects of juglone on liver injury were comparable to those of silymarin. These two groups exhibited similar smoothness of the surface of the liver.

### Histopathological evaluation of the rat livers following drug treatment

**Figure 6:**
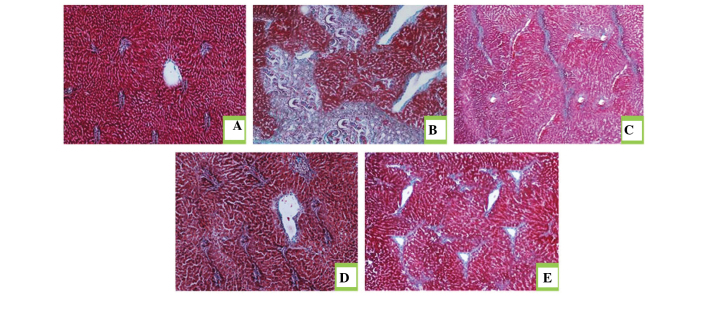
Histopathological sections of liver tissues obtained from various experimental groups. Tissues from the (A) normal control group exhibited normal liver architecture; (B) DMN control group exhibited proliferation of the bile duct, thick fibrous septa and collagen fibers; (C) juglone prevention group exhibited minimal fibrous septa and collagen fibers; (D) silymarin prevention group exhibited marginal fibrous septa and irregular rejuvenating nodules and (E) juglone + silymarin prevention group exhibited insignificant fibrous septa and collagen fibers. Masson's trichrome staining was used and visualized at an original magnification of x20. DMN, dimethylnitrosamine.

Masson's trichrome staining was performed to estimate the extent of fibrosis following treatment with DMN. As shown in Fig. 6A, histological analysis of the rat liver sections revealed that those from the normal group exhibited no collagen deposition. The DMN-treated model groupexhibited proliferation of the bile duct with thick fibrous septa and increased deposition of collagen fibers around the clogged central vein, indicative of significant fibrosis (Fig. 6B). Theliver sections from the rats in the JP group exhibited fewer fibrous septa and uneven regenerating nodules (Fig. 6C). The protective effect of silymarin on liver fibrosis was observedin the SP group (Fig. 6D), treated with silymarin as a standard drug for liver fibrosis. The collagen deposition patterns appeared comparable between the JP group, SP group and JP+SP (Fig. 6E) group. These observations demonstrated the hepatoprotective effect of juglone on hepatic fibrosis in rats.

### Immunohistochemical analysis of juglone treatment revealing decreased expression levels of α-SMA and Col III in the liver

**Figure 7:**
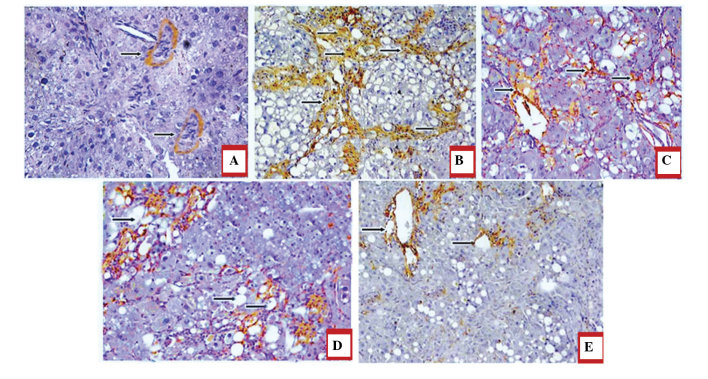
Effect of juglone on the protein expression of α-SMA in the rat liver. The images show representative liver α-SMA immunohistochemical staining in the (A) normal control group, (B) dimethylnitrosamine-induced hepatic fibrosis group, (C) juglone prevention group, (D) silymarin prevention group and (E) juglone + silymarin prevention group. Counterstained with hematoxylin (blue). Original magnification x400. Arrows indicate positive cytoplasmic staining.

The effect of treatment with juglone on the expression ofα-SMA is shown in Fig. 7A–E. Since the expression levels of α-SMA and CoI III are markers for liver fibrosis, the present study investigated the effect of juglone on the expression levels of α-SMA and CoI III. The results of the immunohistochemical staining demonstrated that, in the normal group, α-SMA-positive staining was limited to the vascular walls in the central veins and portal areas, whereas significantly marked immune staining was observed in the central veins, portal areas and surrounding the bile ductules in the DMN-treated model group. In the JP, SP and JP+SP groups, the expression levels of α-SMA were considerably decreased, compared with those in the model group (P<0.05; Fig. 7).

**Figure 8:**
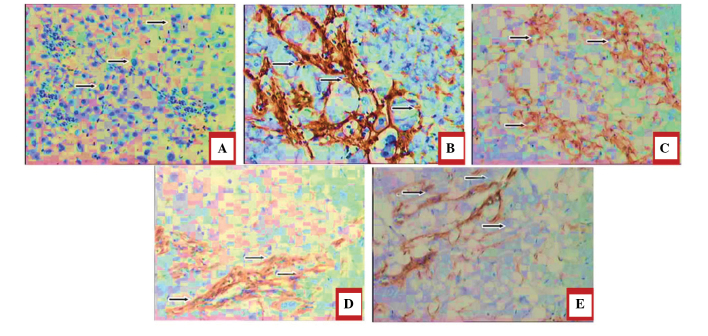
Effect of juglone treatment on the protein expression of collagen III in the rat liver. The images show representative liver collagen III immunohistochemical staining in the (A) normal control group, (B) dimethylnitrosamine-induced hepatic fibrosis group, (C) juglone prevention group, (D) silymarin prevention group and (E) juglone+silymarin prevention group. Counterstained with hematoxylin (blue). Original magnification x400. Arrows indicate positive cytoplasmic staining.

In terms of the expression levels of Col III, an increase in the numbers of Col III positive fibers were observed in the hepatic sinusoids and periportal areas in the model group, whereas a lower expression level of Col III was identified in the normal control group. In the JP, SP and JP+SP treatment groups, the expression levels of Col III were also significantly decreased, compared with the levels in the model group (P<0.05; Fig. 8A–E).

### Effect of juglone on the mRNA expression levels of PPAR-γ and α-SMA

**Figure 9:**
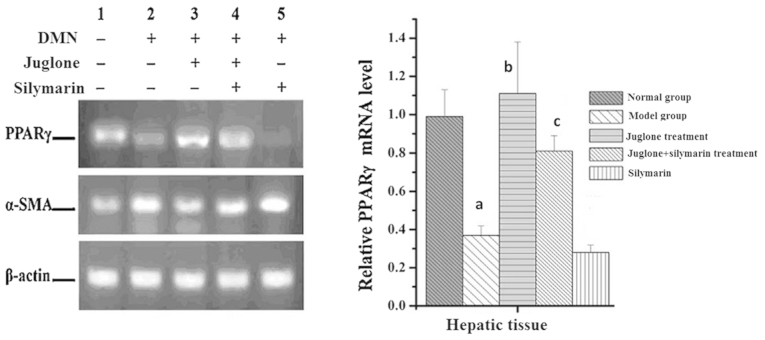
Effect of juglone on the mRNA expression levels of PPAR-γ and α-SMA. The rats were treated with DMN, juglone, silymarin or juglone+silymarin and, after 8 weeks, liver samples were collected. The mRNA expression levels of PPAR-γ and α-SMA in the livers were analyzed and assessed using reverse transcription-quantitative polymerase chain reaction and agarose gel electrophoresis with ethidium bromide. The data are presented as the mean ± standard deviation (n=6). ^a^P<0.01, vs. normal group; ^b^P<0.01, vs. model group; ^c^P<0.05. SMA, smooth muscle actin; PPAR, peroxisome proliferator-activated receptor, DMN, dimethylnitrosamine.

As shown in Fig. 9, treatment with DMNsignificantly suppressed the mRNA expression of PPAR-γ and increased the mRNA expression of α-SMA in the liver tissues (P<0.01). Compared with the model group, silymarin promoted the effect of DMN on the mRNA expression levels of PPAR-γ and α-SMA. By contrast, juglone increased the mRNA expression of PPAR-γ and reduced the mRNA expression of α-SMA, compared with the model and silymarin groups (P<0.01; Fig. 9).

## Discussion

Hepatic fibrosis is a common pathological finding in liver cirrhosis and liver cancer, and is characterized by liver function failure (24). The ECM is largely comprised of collagen proteins, and ECM components, including HA, LN, PCIII and CIV, may be altered by metabolic collagen, which parallels with the degree of hepatic fibrosis (25). Raised serum levels of ALT and AST, triggered by chemical hepatotoxicity, are accompanied by hepatic structural lesions, resulting in functional liver enzymes, which are abnormally present in the cytoplasm and are released into the circulation (26,27). Therefore, elevated levels of liver enzymes are a mark of liver damage, are important for diagnosis by clinicians and are a reflection of liver function (28).

Oxidative stress, as an important primary factor, has been extensively investigated in an increasing number of liver diseases (29). Previous studies have demonstrated that hepatic failure is associated with the production of ROS, collagen synthesis and cellular proliferation, due to oxidative stress, which aggravates inflammation and induces the pathogenesis of hepatic fibrosis (30–32). Oxidative stress augments liver fibrosis through the activation of HSCs, and lipid peroxidationtriggers transcription of the collagen gene. The expression of α-SMA is a representative feature of activated HSCs and this is considered a hallmark for liver fibrosis (33). HSCs are activated following liver damage and segregate into myofibroblast-like cells, which proliferate and contribute to collagen accumulation in the ECM (34). Collagen is responsible for ~55% of the total protein in fibrous liver tissues (35).

The present study demonstrated that juglone markedly decreased the Col III content and decreased the expression levels of α-SMA and CoI III in the liver, indicating an inhibitory effect on the activation of HSCs. It was also demonstrated that juglone increased the activity of SOD and decreased oxidative stress in the liver. These data suggested that the protective effect of juglone in fibrosis may be due to its antioxidative effect in theliver. The results also indicated that juglone markedly increased the speed of recovery of the liver damage. Liver fibrosis caused hepatomegaly and treatment with juglone significantly prevented the effect of DMN on the liver.

DMN increases the levels of serum biochemical parameters, including ALT and AST, which is in accordance with the extent of liver damage (36), and the increase in AST and ALT reflects hepatocellular injury. Treatment with juglone markedly reduced the increased levels of AST and ALT. Following treatment with juglone, the serum enzymatic levels and visceral indices wereconsiderably decreased, signifying that juglone improved liver function and immunocompetence in the DMN-injured rats. The use of Masson's trichrome staining enabled observation and analysis of the developmentof liver fibrosis. The results revealed that treatment with juglone efficiently reversed the liver fibrosis processes in the presence of DMN.

Histological assessment indicated a protective effect of juglone against DMN-induced liver fibrosis. A normal liver has a regular and even surface, but in liver fibrosis it appeared rough and nodular with micronodule and macronodule formation. In the histopathological evaluation, severe structural damage, formation of dense fibrotic septa and proliferation of bile ducts in the presence of inflammatory cells were observed. Treatment with silymarin and juglone recovered the liver structure from fibrosis.

In conclusion, the present study established that the protective effect of juglone in liver fibrosis was associated with increased activity of SOD, reduced oxidative stress and decreased levels of α-SMA and Col III in the liver. Histological data demonstrated that juglone slowed the progression liver fibrosis. The results also demonstrated that the serum levels of ALT, AST, HA, LN, PCIII and CIV were significantly reduced following treatment with juglone. These findings suggested that juglone increased the antioxidative ability of the liver by increasing the activity of SOD, which decreased the ECM collagen accumulation in the liver.
